# Optical and Geometrical Properties from Terahertz Time-Domain Spectroscopy Data

**DOI:** 10.3390/ma17235854

**Published:** 2024-11-29

**Authors:** George Youssef, Nha Uyen T. Huynh, Somer Nacy

**Affiliations:** 1 Experimental Mechanics Laboratory, Mechanical Engineering Department, San Diego State University, 5500 Campanile Drive, San Diego, CA 92182, USA; 2 Biomedical Engineering, University of Baghdad, Baghdad 10071, Iraq

**Keywords:** terahertz time-domain spectroscopy, data extraction, material parameters

## Abstract

Terahertz waves are nondestructive and non-ionizing to synthetic and natural materials, including polymeric and biological materials. As a result, terahertz-based spectroscopy has emerged as a suitable technique to uncover fundamental molecular mechanisms and material properties in this electromagnetic spectrum regime. In terahertz time-domain spectroscopy (THz-TDS), the material’s optical properties are resolved using the raw time-domain signals collected from the sample and air reference data depending on accurate prior knowledge of the sample geometry. Alternatively, different spectral analysis algorithms can extract the complex index of refraction of optically thick or optically thin samples without specific thickness knowledge. A THz-TDS signal without apparent Fabry–Pérot oscillations is commonly associated with optically thin samples, whereas the terahertz signal of optically thick samples exhibits distinct Fabry–Pérot oscillations. While several extraction algorithms have been reported *a priori*, the steps from reducing the time-domain signal to calculating the complex index of refraction and resolving the correct thickness can be daunting and intimidating while obscuring important steps. Therefore, the objective is to decipher, demystify, and demonstrate the extraction algorithms for Fabry–Pérot-absent and -present terahertz signals for various polymers with different molecular structure classifications and nonlinear optical crystal zinc telluride. The experimental results were in good agreement with previously published values while elucidating the contributions of the molecular structure to the stability of the algorithms. Finally, the necessary condition for manifesting Fabry–Pérot oscillations was delineated.

## 1. Introduction

Interest in utilizing the terahertz (THz) regime of the electromagnetic spectrum has peaked during the past three decades within a broad range of scientific and technological applications. Terahertz waves (i.e., light with a wavelength ranging between 30 µm and 3 mm) are found in several domains, including chemical analysis and material science, security and counterfeit detections, food processing and pharmaceuticals, and telecommunication, to name a few prominent examples [1,2,3,4,5,6,7]. For example, in chemical analysis, polymers and ceramics are transparent or semi-transparent to light waves within the THz regime; thus, propagating waves can interrogate molecular vibrations associated with the structure of these materials. In addition, materials scientists have recently foreseen the benefits of THz waves in experimental mechanics and nondestructive evaluations [8,9,10], invigorating new research directions for this non-ionizing, non-invasive, and low-energy region of the electromagnetic spectrum.

At the essence of conventional THz-based spectroscopy, imaging, and nondestructive evaluation experimental techniques is a time-domain spectroscopy setup, irrespective of the physical principles of THz generation and detection. While THz generation and detection are worthy areas for further investigation, attracting prominent researchers worldwide [11,12], the time-domain signal data reduction step can be daunting, especially for newcomers and novice researchers. Hence, the motivation and focus of the research led to this paper.

The attributes of a measured time-domain THz signal are strongly coupled to the THz light-matter interactions, the scanning conditions (e.g., relative humidity and scanning speed), the optical (i.e., the complex refractive index) and electrical properties of the samples, and the geometry of the sample [13,14,15,16,17]. When focusing on the spectral peaks for chemical analysis, as in spectroscopy, calculating the frequency-transformed transfer function from the sample-transmitted (or reflected) and sample-free signals is sufficient to reveal absorption peaks due to molecular vibrations or conformational changes. However, additional calculations are required to resolve the optical properties of the sample, where the sample thickness plays a crucial role in the data reduction stage, resulting in Fabry–Pérot oscillations due to the internal reflections. The Fabry–Pérot oscillations might be visually absent from the signal if the optical thickness is relatively small, concealing the oscillations. In the latter, the main characteristic is the THz peak, and the internal reflections appear indistinguishable.

The general data reduction approach for properties extraction hinges on regressing the signal into a mathematical function containing the components of the complex refractive index. Hence, the properties extraction algorithms can directly operate on the time-domain waveforms or be based on the frequency-transformed signals [17]. In 1996, Duvillaret et al. introduced the analysis technique using different algorithms to extract the complex index of refraction of transmitted THz signals for optically thick and optically thin samples [18]. It should be noted that the echoes of the optically thin counterparts were not observed in the THz signals. This is not to say that internal reflections do not exist in optically thin samples; instead, the distance between the successive echoes is slight. Therefore, the echoes become superimposed and indistinguishable from the prominent THz peak. For optically thick samples, Duvillaret et al. simplified the solution by temporally windowing the THz pulse, effectively ignoring the oscillations in the signal, and removing the Fabry–Pérot term from the complex transmission coefficient of the sample [18]. On the other hand, due to the superimposition of the echoes, windowing the main peak of the signals for optically thin samples would give inaccurate results.

The same research group (i.e., Duvillaret and collaborators) later observed that the frequency-transformed signals of optically thin samples exhibited artificial resonance peaks, which differed in amplitude due to varying estimated thickness values [19]. At the actual (or correct) thickness, these peaks disappeared. Therefore, by recording the amplitude of the peaks as a function of estimated thickness, Duvillaret et al. developed an algorithm to simultaneously resolve the optical properties and sample thickness using THz time-domain spectroscopy measurements [19]. Dorney et al. noted that the accuracy of Duvillaret’s method for optically thick samples relies on the initial thickness estimation [20]; an estimation that is not relatively near the actual thickness can lead to inaccurate results. Then, the total variation method to measure the smoothness of the extracted complex refractive index function was introduced to precisely determine the actual thickness and optical constants of optically thick samples without prior knowledge of the sample geometry [20]. However, the total variance algorithm is not suitable for optically thin samples; hence, the quasi-space (QS) algorithm was introduced by Scheller et al. [21]. Recall that the amplitude of the artificial resonance peaks found in the frequency-transformed signals of samples with an estimated thickness equivalent to the actual thickness is nearly zero. The QS algorithm exploits this attribute by taking the maximum frequency transform of the peaks over a range of estimated thicknesses; here, the minimum value corresponds to the actual thickness [21]. While these methods were previously reported, ambiguous details are essential for successful implementation. The following sections focus on translating some of the above algorithms into quick implementation while providing insights into the physics associated with the mathematics of THz signal processing and analysis.

This paper aims to decipher the existing THz time-domain spectroscopy data reduction approaches based on frequency transformation for calculating the index of refraction and detecting the correct sample thickness. The algorithms presented below generally follow the work of Duvillaret et al. [19]; several improvements and simplifications were revealed to accelerate convergence and demystify data analysis of THz signals.

## 2. Properties Extraction Algorithms

**Figure 1:**
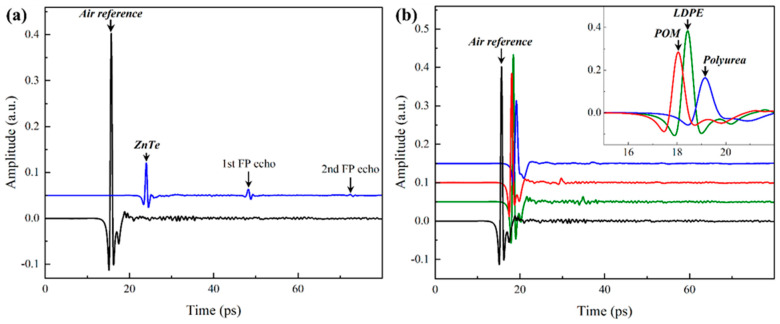
Typical time-domain signals (**a**) with and (**b**) without Fabry–Pérot (FP) oscillations. The signals were vertically shifted for better visualization.

This section is divided into two subsections, each delineating a different algorithm for analyzing THz time-domain spectroscopy signals with apparent and absent Fabry–Pérot oscillations. As mentioned above, the Fabry–Pérot oscillations are manifested in the time domain (e.g., Figure 1a) as secondary or tertiary peaks corresponding to internal reflections based on the optical thickness of the sample and the delay length of the physical THz-TDS setup used in the experiment. Alternatively, the Fabry–Pérot oscillations may be concealed (e.g., Figure 1b) if the optical thickness is relatively small, so the internal reflections are indistinguishable from the main THz peak.

### 2.1. Signals Without Apparent Fabry–Pérot Oscillations

Figure 1b shows typical THz time-domain signals collected using a THz-TDS setup; details of the experimental setup are listed in [9]. Figure 1b also plots a sample-free reference signal using the same scanning parameters and conditions. Three polymeric materials were characterized, including low-density polyethylene (LDPE), polyurea (PU), and polyoxymethylene (POM). Each sample was scanned three consecutive times, preceded by a sample-free reference scan. The following algorithm relies on an initial guess of the mechanical thickness of the samples, which are 1605.5 ± 47.1 µm, 1638.5 ± 13.7 µm, and 1053.4 ± 7.4 µm, respectively. The mechanical thicknesses of the samples were measured using a PosiTector 6000-1 (DeFelsko, Ogdensburg, NY, USA) coating thickness gauge.

The time-domain data of the sample and the reference signals were first transformed to the frequency domain using the fast Fourier transform to calculate the experimental transfer function ($H_{exp}$). The transfer function was then calculated as the ratio of the transformed sample spectrum, $E_{s}\left(\omega \right)$, and the transformed reference spectrum, $E_{r}\left(\omega \right)$, as shown in Equation (1).

(1) Hexp=EsωErω

The magnitude and phase of the experimental transfer function along with the sample mechanical thickness, can be also measured using a micrometer or caliper, were used to estimate the initial components of the complex refractive index

(2a) $$n_{o}=n_{m}-\frac{\mathrm{Arg}\left(H_{exp}\right)c}{2\pi fl_{m}},$$

(2b) $$k_{o}=-\left(\frac{c}{2\pi fl_{m}}\right)ln\left(\left|H_{exp}\right|\right),$$

where, $n_{m}$ is the media index of refraction (assigned unity herein since air was the surrounding media during THz scanning), $l_{m}$ is the mechanical thickness of the sample, f is the frequency, and c is the speed of light. These initial estimations of the sample parameters (e.g., index and thickness) were fed into the following algorithm, searching for the correct index values at the correct thickness iteratively.

The general form of the theoretical transfer function ($H_{th}$) based on the transmission of THz waves through a sample with an arbitrary complex refractive index ($n_{c}=n-ik$) and mechanical thickness ($l_{m}$) is

(3) $$H_{th}=\frac{4n_{c}}{{\left(n_{c}+1\right)}^{2}}\exp\left(\frac{-i\;2\pi fl_{m}\left(n_{c}-1\right)}{c}\right)\sum_{g=0}^{\infty }{\left(\frac{1-n_{c}}{1+n_{c}}\right)}^{2g}\exp\left(\frac{-i\;4\pi fl_{m}n_{c}g}{c}\right),$$

where, g is the number of Fabry–Pérot echoes that may be superimposed on the time-domain data. It is worth reiterating that the internal reflection oscillations are imperceptible for the above reasons. The first two terms of Equation (3) are derived from the transmission and propagation Fresnel coefficients. The third and final term in Equation (3), i.e., the summation term, is the internal reflections due to the Fabry–Pérot oscillations as the THz waves reflect off the internal surfaces of the sample. The derivation of $H_{th}$ is summarized in the Supplementary Materials document.

**Figure 2:**
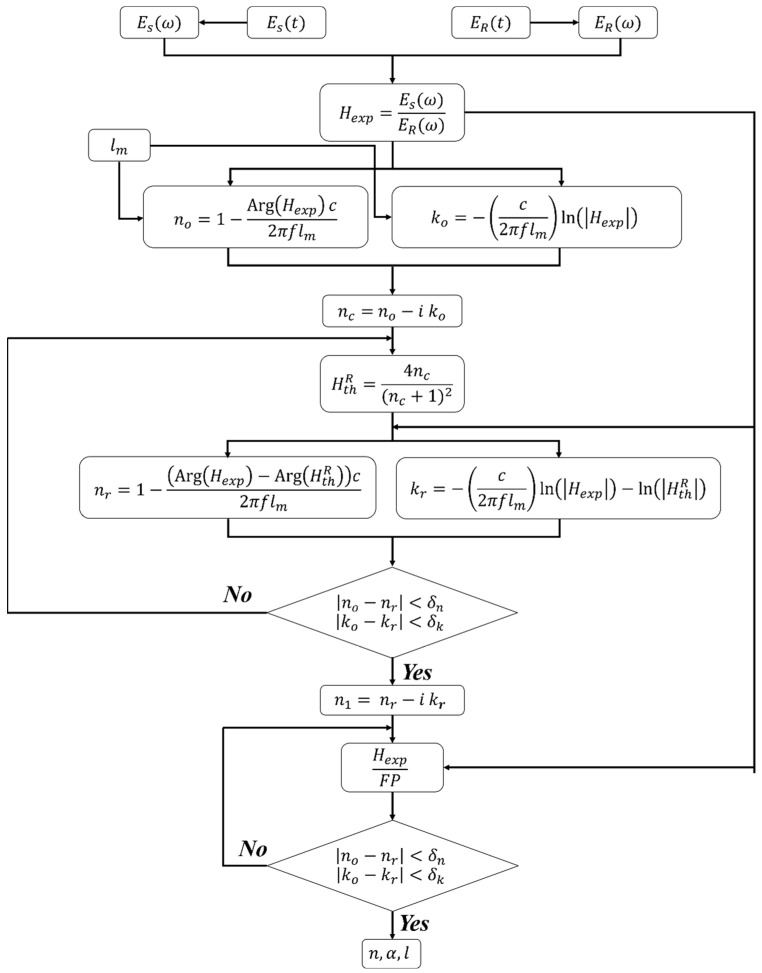
Step-by-step diagram of the parameter extraction algorithm from THz signals without apparent Fabry–Pérot (FP) oscillations.

The initial estimations of the sample parameters were iteratively adjusted to minimize the error between the calculated transfer function, Equation (3), and its measured counterpart, Equation (1). The error minimization can be performed independently on the real and imaginary parts of the refractive index since the transfer functions are complex numbers. Alternatively, it has been suggested previously that the error can be separately minimized between the magnitude and phase of the theoretical and experimental transfer functions [22]. Conceptually, the error minimization approaches are effectively the same since the phase calculates the real part of the index (e.g., Equation (2a)) while the magnitude is part of Equation (2b). The remaining steps of this extraction algorithm are summarized and demonstrated in the step-by-step diagram in Figure 2. The following steps are repeated over a range of assumed thickness centered around the mechanical thickness. The analyst defines the thickness range based on prior knowledge of the accuracy of the measurement tools. Otherwise, an initial wide range of thicknesses can be first considered, followed by a narrower range to reduce the computational cost. That is to say, the accurate knowledge of the initial mechanical thickness is not a prerequisite for precise extraction as an arbitrary thickness range results in solution divergence, i.e., disqualifying the thickness selection.

Step 1: Select a range of thicknesses centered around the initial mechanical thickness ($l_{m}$). At each thickness value, calculate the corresponding initial estimation of the components of the index of refraction (Equation (2)) based on the magnitude and phase of the experimental transfer function.

Step 2: Calculate the reduced theoretical transfer function by ignoring the Fabry–Pérot oscillations and propagation terms such that

$$H_{th}^{R}=\frac{4n_{c}}{{\left(n_{c}+1\right)}^{2}},$$

and use its magnitude and phase to calculate a revised estimation of the real ($n_{r}$) and imaginary ($k_{r}$) parts of the index using Equation (2). In doing so, the $\mathrm{Arg}\left(H_{exp}\right)$ term in Equation (2a) is replaced by $\mathrm{Arg}\left(H_{exp}\right)-\mathrm{Arg}\left(H_{th}^{R}\right)$ while a new $\ln\left(\left|H_{exp}\right|\right)-ln\left(\left|H_{th}^{R}\right|\right)$ term substitutes $ln\left(\left|H_{exp}\right|\right)$ in Equation (2b). These substitutions effectively reintroduce the propagation term that was previously neglected. The revised estimations are then iteratively calculated until the convergence criteria are reached, at which point, the next step of the algorithm commences. Notably, this final sub-step was carried out by overusing the Nelder–Mead method to accelerate convergence, a need that was not substantiated herein since the following convergence approach was performed efficiently.

$$\left|n_{o}-n_{r}\right|<\delta_{n}\;and\;\left|k_{o}-k_{r}\right|<\delta_{k}$$

Step 3: The approach in the final step mimics the procedure outlined in Step 2 but after eliminating the Fabry–Pérot effect from the experimental transfer function. This is done by dividing $H_{exp}$ by the Fabry–Pérot oscillations while assuming an infinite number of echoes (i.e., g→∞) in Equation (3). The division by the Fabry–Pérot term effectively accounts for the final term in Equation (3), which was previously neglected in the calculation of the reduced transfer function.

$$FP=\frac{1}{1-{\left(\frac{1-n_{c}}{n_{c}+1}\right)}^{2}\exp\left(\frac{-i\;4\pi fl_{m}\left(n_{c}\right)}{c}\right)}.$$

The complex index of refraction from Step 2 is used as the initial guess for the calculation. The new index components are then calculated by minimizing the difference between the current values and those from the previous iteration.

Only at the correct thickness are the refractive index components nearly free of oscillations. Therefore, the difference between the average peak and valley values, i.e., the amplitude of the superimposed oscillations, is used to identify the optimal and correct thickness [19].

### 2.2. Signals with Fabry–Pérot Oscillations

**Figure 3:**
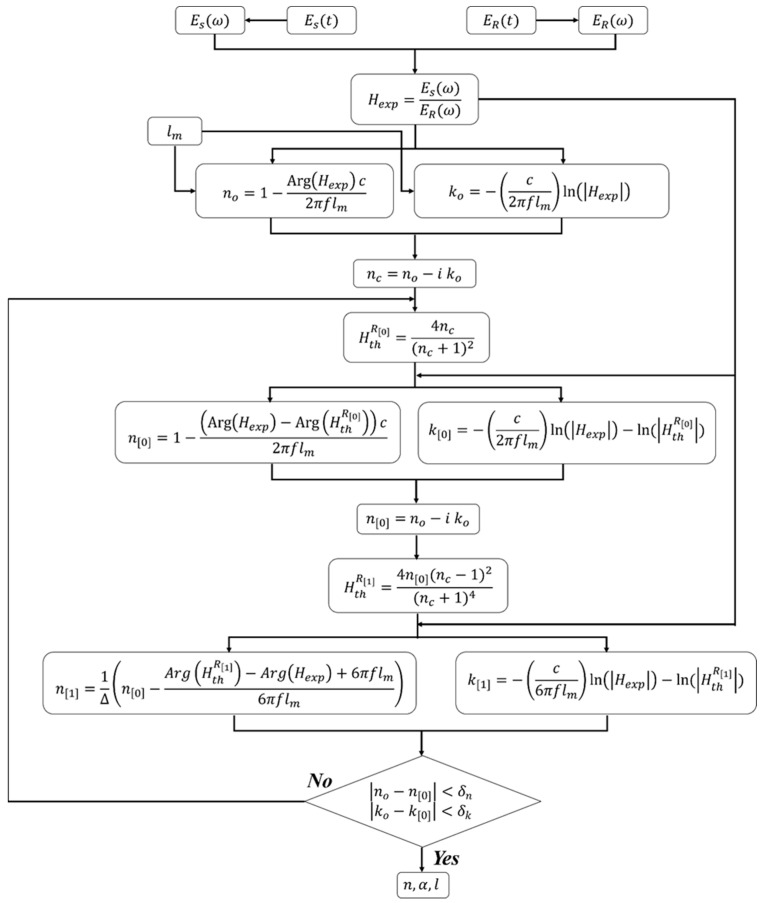
Step-by-step diagram for the properties extraction algorithm from THz-TDS time-domain data with apparent Fabry–Pérot (FP) oscillations.

Figure 1a is a typical THz time-domain signal with apparent Fabry–Pérot echoes, requiring a different analysis protocol than that discussed above. However, there are a few overlapping steps with the data reduction algorithm of signals without Fabry–Pérot oscillations. Similar to the algorithm above, the experimental transfer function is first calculated using Equation (1), followed by initial estimations of the refractive index components using the magnitude and phase of $H_{exp}$, as shown in Equation (2), while assuming a range of thicknesses centered around the mechanical thickness of the sample. The remaining steps emphasize the analysis of the frequency spectrum of $H_{exp}$ by only considering the first and main THz peak (i.e., g=0 in Equation (3)) to extract refractive index components. The latter is done based on the time shift between the reference sample-free signal and the first THz peak in the sample signal. The second step accentuates the extraction of the complex refractive index based on the second peak in the signal (i.e., 1^st^ Fabry–Pérot echo such that g=1 in Equation (3)). Of course, this can be done by considering any other Fabry–Pérot echo while applying the appropriate modification to the algorithm, e.g., adjusting the delay time. The algorithm for analysis of time-domain signals with apparent Fabry–Pérot oscillations is summarized and succinctly illustrated in Figure 3.

Step 1: Select a range of thicknesses near the initial mechanical thickness ($l_{m}$) and calculate the corresponding initial estimation of the components of the index of refraction (Equation (2)).

Step 2: Calculate the reduced theoretical transfer function ($H_{th}^{R_{\left[0\right]}}=\frac{4n_{c}}{{\left(n_{c}+1\right)}^{2}}$) and revise the estimation of the real ($n_{\left[0\right]}$) and imaginary ($k_{\left[0\right]}$) parts by only considering the main THz peak. In doing so, the $\mathrm{Arg}\left(H_{exp}\right)$ term in Equation (2a) is replaced by $\mathrm{Arg}\left(H_{exp}\right)-\mathrm{Arg}\left(H_{th}^{R_{\left[0\right]}}\right)$ while $\ln\left(\left|H_{exp}\right|\right)-ln\left(\left|H_{th}^{R_{\left[0\right]}}\right|\right)$ substitutes $ln\left(\left|H_{exp}\right|\right)$ term in Equation (2b). Similarly, the revised estimations are then iteratively calculated until the convergence criteria are reached, at which point, the next step of the algorithm commences.

$$\left|n_{o}-n_{\left[0\right]}\right|<\delta_{n}\;and\;\left|k_{o}-k_{\left[0\right]}\right|<\delta_{k}$$

Step 3: The first Fabry–Pérot echo is now considered to calculate another form of the theoretical transfer function

$$H_{th}^{R_{\left[1\right]}}=\frac{4n_{c}{\left(n_{c}-1\right)}^{2}}{{\left(n_{c}+1\right)}^{4}}.$$

The magnitude and phase of $H_{th}^{R_{\left[1\right]}}$ are then used to calculate the complex refractive index based on the 1^st^ Fabry–Pérot echo by revising the definitions in Equation (2). Here, the complex index of refraction from Step 2 is used as the initial guess for the calculation herein. The new index components are

$$n_{\left[1\right]}=\frac{1}{∆}\left(n_{\left[0\right]}-\frac{Arg\left(H_{th}^{R_{\left[1\right]}}\right)-Arg\left(H_{exp}\right)+6\pi fl_{m}}{6\pi fl_{m}}\right),$$

$$k_{\left[1\right]}=-\left(\frac{c}{6\pi fl_{m}}\right)ln\left(\left|H_{exp}\right|\right)-ln(\left|H_{th}^{R_{\left[1\right]}}\right|),$$

where, ∆ is the time delay ratio of the 1^st^ Fabry–Pérot echo with respect to the reference and main sample THz peak. The mathematical form of ∆ is derived in the Supplementary Materials document while considering the time delay between the main THz peak, the 1^st^ Fabry–Pérot echo, and the reference, sample-free peak. The iterative convergence is terminated according to the same rationale as discussed in the previous section.

## 3. Results and Discussion

**Figure 4:**
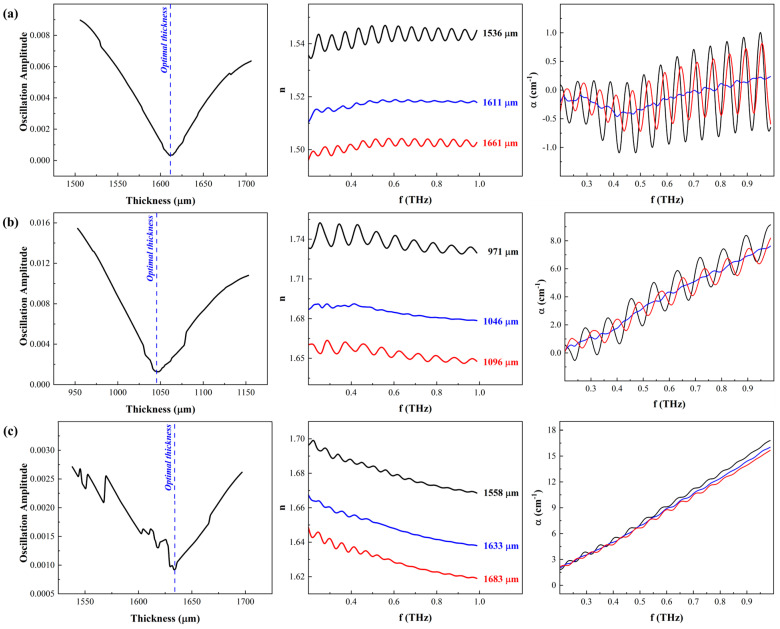
A composite of the results, thickness, real index, and absorption coefficient, based on applying properties extraction algorithm outlined in Section 2.1 on THz-TDS signals from (**a**) low-density polyethylene (LDPE), (**b**) polyoxymethylene (POM), and (**c**) polyurea (PU).

Figure 4 is a composite chart of the oscillation amplitude as a function of examined thicknesses, the real refractive index, and the absorption coefficient as a function of the frequency for three polymers. The THz time-domain signals of these polymers did not exhibit any apparent Fabry–Pérot oscillations. As mentioned above, only one optimal combination of the correct thickness and the refractive index yields spectra with suppressed facetious oscillations on the real and imaginary components. That is to say, the iterative selection of the sample thickness that results in the oscillation-free refractive index and absorption coefficient of the material as a function of frequency is considered the actual correct thickness. Hence, the difference between the peaks and the valleys of the oscillations, i.e., the amplitude of oscillations superimposed on the index spectra, was calculated at each assumed thickness, where the minimum difference corresponds to the correct thickness. The latter is then used to extract the components of the refractive index without notable oscillations (or notably reduced compared to other thicknesses) since the complete removal of the oscillations depends not only on the convergence to the correct thickness but also on the absorption coefficient, i.e., a function of the molecular structure of the polymer, as discussed later. For example, Figure 4a plots the amplitude of the oscillations (V-shaped plot in the left panel), the real refractive index at three different thicknesses (middle panel), and the absorption coefficient (right panel) for low-density polyethylene. The three representative thicknesses include the optimal or correct thickness, residing at minima of the V-shaped amplitude of the oscillations plot, and two other thicknesses; one is selected below the correct thickness, and the other is above. As a result, the real refractive index and the absorption coefficient plots at the correct thickness are nearly oscillation-free, which is not the case at any other thickness. Figure 4b,c are the same results for polyoxymethylene and polyurea, respectively.

The results reported in Figure 4 affirm three noteworthy observations related to the performance of the properties extraction algorithm and the macromolecule structure. Notably, the previously reported algorithms were generally applied to signals collected from crystalline materials such as LiNbO_3_, high-resistivity silicon, and optical glass [18,19,21], confiscating essential nuances of the analysis. Hence, some of the following observations were not discussed *a priori*. First is the transition sharpness of the amplitude of the oscillations plot as a function of examined thicknesses, pointing to interdependence on the materials. Regardless of the material, the algorithm was applied to a wide range of thicknesses, spanning 200 μm around the mechanical thickness, where the refractive index and amplitude of the superimposed oscillations were calculated. However, the sharpness of the plot delineating the minimum oscillation amplitude and identifying the optimal thickness has evolved from a clear point for LDPE, a valley of proximate values for POM, to scattered values for PU. It is worth noting that irrespective of the final shape of the amplitude vs. thickness graph, the algorithm successfully identified the correct thickness for each sample, which was found to be in the vicinity of and in reasonable agreement with the mechanical thickness. In previous reports, the transition of the oscillation amplitude through the correct sample thickness was distinct and easily identifiable, attributed to the atomic structure of those materials, as mentioned above. Here, the three selected polymers span different molecular classifications and tacticity, where both LDPE and POM are semi-crystalline thermoplastic polymers, while polyurea is a cross-linked thermoset elastomer [23]. Due to their generally linear chains, the semi-crystalline nature of LDPE and POM results in easily identifiable minimum amplitude corresponding to the optimal thickness since their quasi-ordered molecular structures minimize the dispersion of the propagating THz waves. On the other hand, the lack of order in the cross-linked polyurea implies a high probability of wave dispersion and loss of the characteristic attributes of the V-shaped amplitude vs. thickness plot. Table 1 summarizes the thickness, the real refractive index, and the maximum absorption coefficient of these materials based on the frequency analysis of THz-TDS time-domain signals.

**Figure 5:**
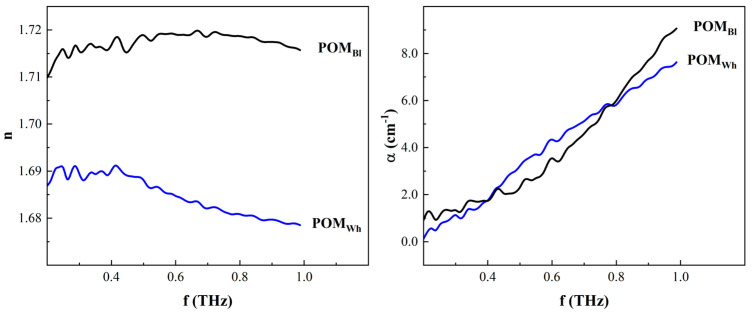
Comparison of the refractive indexes and absorption coefficients of black (POM_Bl_) and white (POM_Wh_) polyoxymethylene at the corresponding optimal thicknesses.

Second, the real refractive index values of three characterized polymers are 1.52 ± 0.01, 1.68 ± 0.01, and 1.65 ± 0.01 for LDPE, POM, and PU, respectively. The refractive index of these polymers as a function of frequency are plotted in Figure 4a–c, respectively (middle panels), showing nearly oscillation-free refractive indexes at the correct thickness reported in Table 1. As predicted, the refractive index at any other thickness exhibits pronounced oscillations, indicating convergence to the correct parameters has not yet been achieved. Since the index is inversely proportional to the thickness, higher thicknesses result in underestimation, while lower thickness overestimates the index. The index values for LDPE and PU agreed with previous publications [10,24]; however, the POM index values reported herein are significantly higher than those reported in [25]. The latter used a similar THz-TDS setup to that used in the research leading to this paper. The discrepancy between the values herein and those in [25] is attributed to the source and color of the POM samples. The POM used in this study was industrial grade, acquired from a commercial supplier, but the source of the samples used in [25] was not explicitly disclosed; hence, it is assumed to be research grade. The source indicates possible changes in the manufacturing conditions, which might result in significant molecular structure changes, such as density changes due to different degrees of crystallization [23]. The latter is a significant factor in the values of the refractive index, according to the Lorenz–Lorentz and Dale–Gladstone laws [26,27]. The natural color of POM is known to be colorless, transparent beads, which may become opaque as a function of degree of crystallinity or colorful by adding different pigmentations or colorants. The POM samples used herein were white, implying the possibility of either a different degree of crystallization or intentional coloring. The color of the POM in [25] was not reported. To verify the effect of color on the index, a black POM sample (here, it is apparent a pigment was used given the natural color of the polymer) was characterized using the same setup and analyzed using the algorithm outlined in Section 2.1. Figure 5 compares the white and black POM index, where the latter was higher than the former, demonstrating the correlation between pigmentation and refractive index in the THz regime. This is not surprising since THz-TDS has recently proliferated several industrial applications for quality control and quality assurance, given its sensitivity to the presence of foreign chemicals and containments [13,14,15].

Finally, the values of the absorption coefficients (right panels in Figure 4) of the three investigated polymers substantiate the differences in the amplitude oscillation plots (left panels in Figure 4). The absorption coefficient of polyurea was the highest compared to the LDPE and POM counterparts, where LDPE reported the lowest absorption coefficient. The relatively low attenuation of LDPE resulted in a sharp inflection point in the amplitude oscillation plot and a nearly steady refractive index within the investigated range of frequencies. As the frequency increased, the moderate absorption of POM yielded a mild descending trend in the refractive index. It also resulted in a slight bluntness of the V-shaped plot of the amplitude oscillations, as shown in the left panel of Figure 4b. The high absorption coefficient of polyurea similarly affected the refractive index, which continued to decrease as the frequency increased since the attention degraded the strength of the real index. As mentioned earlier, the cross-linked molecular structure is thought to be related to the increased absorption coefficient since the molecular order in polyurea is inferior to the semi-crystalline LDPE and POM.

**Figure 6:**
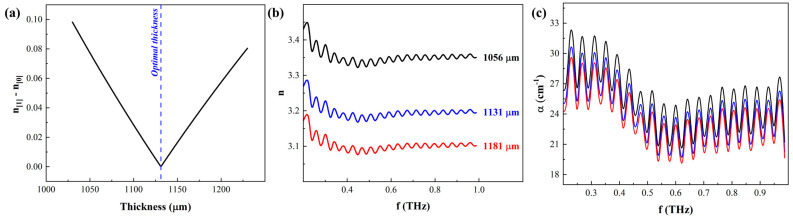
Resulting (**a**) optimal thickness, (**b**) refractive index, and (**c**) absorption coefficient of ZnTe based on the algorithm using THz signals with apparent Fabry–Pérot echoes.

Figure 6 shows the results of analyzing the THz signals of ZnTe using the properties extraction algorithm outlined in Section 2.2. The time-domain data of ZnTe exhibited two apparent Fabry–Pérot echoes (Figure 1a), allowing the extraction of the complex refractive index using the main THz peak and the first Fabry–Pérot echo. While the same process can be extended to use the second Fabry–Pérot echo instead of the first, the signal strength of the former prohibited this approach. In essence, the refractive index extracted from the main peak and any subsequent Fabry–Pérot echoes must be the same if the correct thickness of the sample is accurately identified. Therefore, the algorithm in Section 2.2 was terminated once the indexes extracted from the main THz peak matched that derived from the first Fabry–Pérot echo, i.e., the difference is minimized. Figure 6a plots the difference between the indexes from the main peak and Fabry–Pérot echo, where the minimum difference pinpoints the accurate thickness of the sample. At the latter, the components of the index were extracted and plotted in Figure 6b,c while also plotting the unoptimized index results at two other arbitrary thicknesses above and below the correct one. The absorption coefficient in this case was higher than those reported for the polymers above. Still, the crystalline structure assisted in manifesting the Fabry–Pérot oscillations and facilitating the properties extraction. The real index of ZnTe was found to be 3.22 ± 0.06, on average, in excellent agreement with previous reports [28]. The modified algorithm was robust in identifying the accurate sample thickness and the refractive index components. The convergence was guaranteed since incorrect thickness assumption results in a noticeable difference between the index predicted from the main THz peak and the first Fabry–Pérot echo.

At the outset, it is fitting to elucidate the necessary conditions for the manifestation of the Fabry–Pérot oscillations since the algorithms were separated to analyze signals without (Section 2.1) and with (Section 2.2) Fabry–Pérot echoes. It is well known that the THz time-domain signals of materials with refractive index higher than two are guaranteed to have observable Fabry–Pérot oscillations. On the other hand, the signals of materials with indexes of 1<n<2 are commonly oscillation-free, as shown in Figure 1b. The rationale is based on the ratio of the time delay between the main THz peak and the first Fabry–Pérot echo, which is recalled from the Supplementary Materials document to be

(4) $$\Delta =\frac{\Delta_{2}-3\Delta_{1}}{2\Delta_{1}}=\frac{1}{n-1}.$$

The values of Δ→∞ as n→1 while Δ→0 as the refractive index increases. That is to say, the manifestation of Fabry–Pérot oscillations is not conducive to all n<2 since the internally reflected waves chase the primary transmitted THz waves nearby, making them indistinguishable. On the other hand, *n* ≥ 2 provides the necessary conditions within the current physical setup limitations for the begetting of distinguishable Fabry–Pérot echoes. Figure S3 of the Supplementary Materials document provides the corresponding mathematical visualization for this rationale. In all, an approach that may overcome the current limitations is the utilization of femtosecond lasers with higher repetition rates, a topic beyond the scope of this research.

## 4. Conclusions

Data reduction of THz time-domain spectroscopy signals can be overwhelming, posing several challenges to novice analysts, given the difference in algorithms depending on the optical properties of the investigated materials. In the research leading to this report, two algorithms were reintroduced to analyze THz-TDS data with and without apparent internal reflection echoes, e.g., Fabry–Pérot oscillations. In doing so, the removal of the Fabry–Pérot oscillations from optically thin samples, i.e., signal without apparent internal reflection echoes, was used to resolve the optical properties of the material, including the refractive index and absorption coefficient, while simultaneously identifying the correct thickness, at which factious oscillations in the results were suppressed. On the other hand, Fabry–Pérot echoes facilitated the extraction of the optical properties at the correct thickness from optically thick samples based on the convergence of the refractive index extracted from the main THz peak and subsequent Fabry–Pérot echo. Irrespective of the optical condition, the algorithms performed robustly, effectively reporting the refractive index, absorption coefficient, and the correct thickness of the samples, where the results were found in general agreement with previous reports. The results reported herein pointed to the absorption coefficient’s influence, based on the molecular structure of polymers, on the performance of the algorithms. The necessary conditions for manifesting Fabry–Pérot oscillations, delineating the difference between optical thick and optically thin samples, were mathematically derived.

## Figures and Tables

**Table 1:** Summary of the correct thickness, refractive index, and absorption coefficient from the results in Figure 4.

Polymer	Thickness (µm)	n	$\alpha \;({cm}^{-1})$
Low-density polyethylene	1612.3 ± 1.5	1.52 ± 0.01	-
Polyoxymethylene	1053.45 ± 7.4	1.68 ± 0.01	3.99 ± 2.27
Polyurea	1050.3 ± 0.8	1.65± 0.01	8.75 ± 4.21

## Data Availability

The original contributions presented in the study are included in the article/Supplementary Materials, further inquiries can be directed to the corresponding author.

## Supplementary Materials

The following supporting information can be downloaded at: https://www.mdpi.com/article/10.3390/ma17235854/s1. Figure S1: Transmitted terahertz wave propagating through a sample (top panel) and reference media (bottom panel). Figure S2: General terahertz time-domain signals of air reference and sample with apparent Fabry-Pérot oscillations or echoes. Figure S3: Relation of the change in delay times as a function of increasing refractive index values.
