# Supplementary material for: Optical and Geometrical Properties from Terahertz Time-Domain Spectroscopy Data
Source: Materials (Basel). 2024 Nov 29;17(23):5854. doi: 10.3390/ma17235854 (PMC11642474; doi:10.3390/ma17235854)
Supplement: Supplementary file 1 [file materials-17-05854-s001.zip › materials-3283962-supplementary.pdf]

Supplementary Materials

Optical and Geometrical Properties from Terahertz Time-Domain Spectroscopy Data

George Youssef*, Nha Uyen T. Huynh, and Somer Nacy

Experimental Mechanics Laboratory, Mechanical Engineering Department, San Diego State University, 5500 Campanile Drive, San Diego Ca 92182

Keywords: terahertz time-domain spectroscopy, data extraction, material parameters

***Corresponding Author:** gyoussef@sdsu.edu

1. Derivation of the theoretical transfer function

The theoretical transfer function, H_{th}, for a terahertz (THz) time-domain spectroscopy signal can be derived by considering the propagation of a terahertz wave, normal in incidence, through a single layer sample with a thickness l_m and complex index of refraction $n_c = n - ik$. The refractive index of the media surrounding the sample is taken as n_m. The distance between the source of the emitted wave and first surface of the sample and the distance between the second surface of the sample and detector is l_1 and l_2, respectively. Figure S1 shows a schematic of the terahertz wave propagating through a sample (top panel) and the same wave propagating through the reference media (bottom panel). It should be noted that the angle of the wave propagation shown in the top panel of Figure S1 is exaggerated to better visualize the internal reflections within the sample. Physically, there is an angle (θ) between the propagated wave and the plane perpendicular to the surface of the sample due to the change in refractive index between the media and sample. However, when the emitted incident wave is normal to the surfaces of the sample this angle is nearly negligible. Therefore, θ is taken as 0 for the derivation herein.

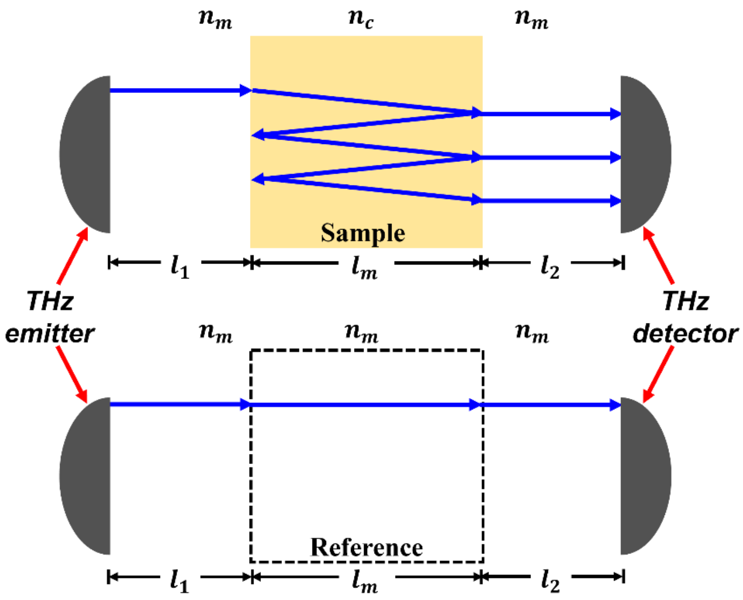

Figure S1: Transmitted terahertz wave propagating through a sample (top panel) and reference media (bottom panel).

When an electromagnetic wave arrives at an interface between two different materials, part of the incident wave is reflected while the other part is transmitted. Fresnel coefficients, Eqns. S1-S4, can then be used to determine the ratio of the incident wave that is reflected and transmitted,

$$R_{ms} = \frac{n_c - 1}{n_c + 1} \tag{S1}$$

$$R_{sm} = \frac{1 - n_c}{n_c + 1} \tag{S2}$$

$$T_{ms} = \frac{2}{n_c + 1} \tag{S3}$$

$$T_{sm} = \frac{2n_c}{n_c + 1} \tag{S4}$$

where, the subscript ms represents the interface at which the wave propagates from the media to the sample, and vice versa for a subscript of sm. In this study, the media surrounding the sample is air; therefore, n_m has been assigned unity for the rest of the derivation and in the main article. As the light waves propagates from the emitter to the detector, the energy of the wave is also absorbed and the amplitude of the signal attenuates based on the speed of light in the media and the sample, the travel distance of the wave in the corresponding medium, and the frequency of the electromagnetic wave (f). In other words, the general propagation coefficient of a wave traveling through a medium with an index of n and travel distance l is

$$P = exp\left(\frac{-i\,2\pi f l n}{c}\right) \tag{S5}$$

where, c is the speed of light in vacuum.

The theoretical transfer function is the ratio between the transmitted electric field when the THz interacts with the sample and the transmitted electric field of the THz in air, $H_{th} = {}^{E_s}/_{E_R}$. From the bottom panel of Figure S1, the reference transmitted electric field can be broken into four terms, the initial electric field originating from the emitter and three propagating terms.

$$E_R = E_I exp\left(\frac{-i\,2\pi f l_1}{c}\right) exp\left(\frac{-i\,2\pi f l_m}{c}\right) exp\left(\frac{-i\,2\pi f l_2}{c}\right) \tag{S6}$$

On the other hand, the transmitted electric field through the sample, E_s, is reduced to the summation of all transmitted electric field branches. For example, the top panel of Figure S1 illustrates the first three transmitted branches because of the THz-matter interaction with the sample. The transmitted electric field after the first branch can be written as $E_I P_{l_1}^m T_{ms} P_{l_m}^s T_{sm} P_{l_2}^m$, here the superscript in each propagation term represents the medium (m) and the sample (s) while the subscripts denote the distance traveled. The total transmitted electric field after the second (Eqn. S7) and third (Eqn. S8) branches are found to be

$$E_I P_{l_1}^m T_{ms} P_{l_m}^s T_{sm} P_{l_2}^m + E_I P_{l_1}^m T_{ms} P_{l_m}^s R_{sm} P_{l_m}^s R_{sm} P_{l_m}^s T_{sm} P_{l_2}^m = E_I P_{l_1}^m T_{ms} P_{l_m}^s T_{sm} P_{l_2}^m \left[1 + {R_{sm}}^2 {P_{l_m}^s}^2\right] \tag{S7}$$

$$E_I P_{l_1}^m T_{ms} P_{l_m}^s T_{sm} P_{l_2}^m \left[1 + {R_{sm}}^2 {P_{l_m}^s}^2 + {R_{sm}}^4 {P_{l_m}^s}^4\right] \tag{S8}$$

Therefore, the total transmitted signal for E_s for an infinite number of branches, where each branch is represented as g, is,

$$E_s = E_I P_{l_1}^m T_{ms} P_{l_m}^s T_{sm} P_{l_2}^m \sum_{g=0}^{\infty} \left(R_{sm} P_{l_m}^s\right)^{2g} \tag{S9}$$

Herein, each branch is referred to a pulse manifested in the terahertz time-domain signal (Figure S2), where any pulse after the main pulse is a Fabry-Pérot (FP) echo, *i.e.*, an internal reflection. From Eqns. S6 and S9, and after substituting the Fresnel and the propagation coefficients (Eqns. S1-S5), the theoretical transfer function is derived to be

$$H_{th} = \frac{4n_c}{(n_c+1)^2} \exp\left(\frac{-i\,2\pi f l_m (n_c-1)}{c}\right) \sum_{g=0}^{\infty} \left(\frac{1-n_c}{1+n_c}\right)^{2g} \exp\left(\frac{-i\,4\pi f l_m n_c g}{c}\right). \tag{S10}$$

Eqn. S10 is recalled in the main document and used in the development of the properties extraction algorithms reported in Section 2.

2. Derivation of the time delay ratio for optically thick samples

It is apparent from Figure S2 that the real value of the refractive index of the main pulse and of the 1st FP echo is $n_{[0]} = \Delta_1 c/l_m + 1$ and $n_{[1]} = \frac{1}{3}(\Delta_2 c/l_m + 1)$, respectively. Δ_1 is the delay time between the reference and main pulse and Δ_2 is the delay time between the reference and 1st FP echo. It should be noted that $n_{[0]}$ and $n_{[1]}$ are equal, as the material properties of the sample does not change during the THz-matter interaction. Therefore, it can be shown that $\Delta_2 - 3\Delta_1 = 2l_m/c$, by rearranging the index equation of the main pulse. The relationship between the delay times and refractive index is the delay time ratio used in the main document

$$\Delta = \frac{\Delta_2 - 3\Delta_1}{2\Delta_1} = \frac{1}{n-1}. \tag{S11}$$

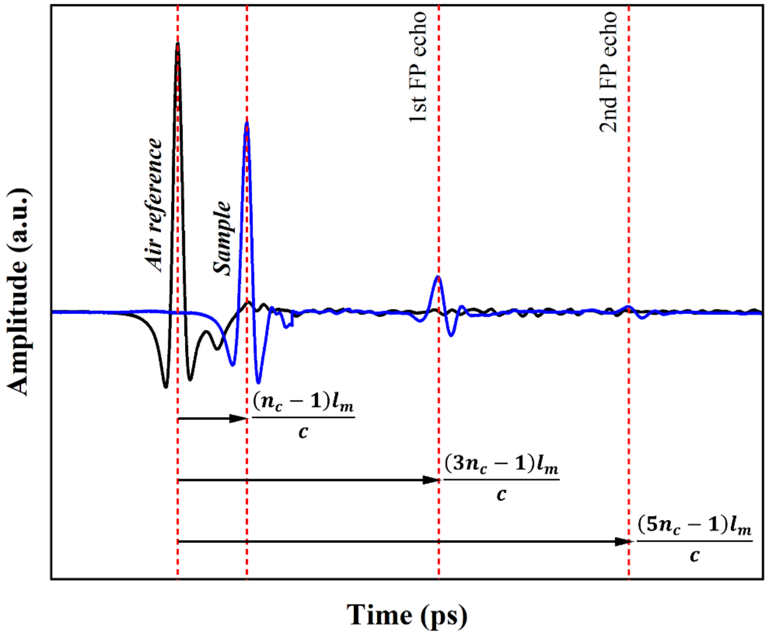

Figure S2: General terahertz time-domain signals of air reference and sample with apparent Fabry-Pérot oscillations, or echoes.

Here, we visualize the behavior of the time delay ratio as a function of possible refractive indexes (i.e., a function of different materials). Figure S3 plots Δ as a function of different n values,

showing the bounds of the values while elucidating the rationale for $n = 2$ for manifesting the Fabry-Pérot in terahertz time-domain spectroscopy signals.

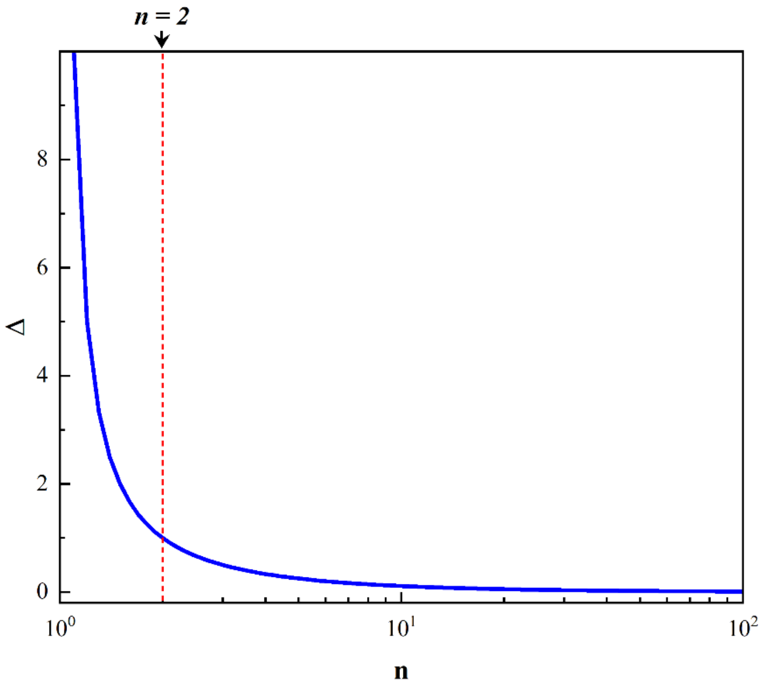

Figure S3: Relation of the change in delay times as a function of increasing refractive index values.
